# Metadensity: a background-aware python pipeline for summarizing CLIP signals on various transcriptomic sites

**DOI:** 10.1093/bioadv/vbac083

**Published:** 2022-11-10

**Authors:** Hsuan-Lin Her, Evan Boyle, Gene W Yeo

**Affiliations:** Bioinformatics and Systems Biology Program, University of California San Diego, La Jolla, CA 92093, USA; Department of Cellular and Molecular Medicine, University of California San Diego, La Jolla, CA 92093, USA; Department of Cellular and Molecular Medicine, University of California San Diego, La Jolla, CA 92093, USA; Bioinformatics and Systems Biology Program, University of California San Diego, La Jolla, CA 92093, USA; Department of Cellular and Molecular Medicine, University of California San Diego, La Jolla, CA 92093, USA; Institute for Genomic Medicine, University of California San Diego, La Jolla, CA 92093, USA; Stem Cell Program, University of California San Diego, La Jolla, CA 92093, USA

## Abstract

**Motivation:**

Cross-linking and immunoprecipitation (CLIP) is a technology to map the binding sites of RNA-binding proteins (RBPs). The region where an RBP binds within RNA is often indicative of its molecular function in RNA processing. As an example, the binding sites of splicing factors are found within or proximal to alternatively spliced exons. To better reveal the function of RBPs, we developed a tool to visualize the distribution of CLIP signals around various transcript features.

**Results:**

Here, we present Metadensity (https://github.com/YeoLab/Metadensity), a software that allows users to generate metagene plots. Metadensity allows users to input features such as branchpoints and preserves the near-nucleotide resolution of CLIP technologies by not scaling the features by length. Metadensity normalizes immunoprecipitated libraries with background controls, such as size-matched inputs, then windowing in various user-defined features. Finally, the signals are averaged across a provided set of transcripts.

**Availability and implementation:**

Metadensity is available at https://github.com/YeoLab/Metadensity, with example notebooks at https://metadensity.readthedocs.io/en/latest/tutorial.html.

**Supplementary information:**

Supplementary data are available at *Bioinformatics Advances* online.

## 1 Introduction

RNA-binding proteins (RBPs) are key modulators of RNA metabolism (Hentze *et al.*, 2018). Cross-linking and immunoprecipitation (IP) followed by sequencing (HITS-CLIP/CLIP-seq) (Darnell, 2010; Licatalosi *et al.*, 2008; Yeo *et al.*, 2009) and derivatives such as PAR-CLIP (Hafner *et al.*, 2010), iCLIP (Briese *et al.*, 2019; König *et al.*, 2010), enhanced CLIP (eCLIP) (Van Nostrand *et al.*, 2016) and irCLIP (Zarnegar *et al.*, 2016) are technologies to discover transcriptome-wide RNA interaction sites of RBPs. Briefly, after crosslinking of the RBP to RNA and limited digestion of unprotected RNA, the protected RBP–RNA fragment is isolated by IP, converted into cDNA and then sequenced. During library preparation, depending on the reverse transcription conditions, the crosslinking of the nucleotide causes reverse transcription stoppage or mutation (Chakrabarti *et al.*, 2018; Hauer et al., 2015; Van Nostrand *et al.*, 2017). As a result, crosslink-induced read truncations (CITs) or mutations (CIMs) can enable near-nucleotide resolution recovery of a fraction of the binding sites.

Enriched RBP binding at specific transcript features provide important clues to the function of the RBP. To illustrate, spliceosomal proteins are enriched at the 5′- and 3′-splice sites (ss) (Moore and Sharp, 1993), and RNA decay factors often interact within the 3′-untranslated regions (UTRs) of protein-coding genes (Muers, 2013). By examining the distribution of RBP-binding sites around canonical features in genes, one can infer the functions of RBPs.

The distribution of transcriptome-wide signals is often summarized in metagene plots. However, existing metagene packages (Olarerin-George and Jaffrey, 2017) emphasize the 5′-UTR-CDS-3′-UTR model on mature messenger RNAs (mRNAs). Such a model is useful in studying RNA stability and/or translational regulators. However, many RBPs bind premature mRNAs to regulate splicing, polyadenylation and export (Hentze *et al.*, 2018). To thoroughly comprehend an RBP’s role in RNA processing, a software tool that includes multiple models of metagene density is needed. In addition, CLIP-seq data contain various background signals (Van Nostrand *et al.*, 2016) and existing metagene packages do not support background normalization. The coverage at each position is strongly influenced by the expression level of the substrate. The use of a size-matched input (SMInput) library in eCLIP accounts for non-specific background signal in the identical size range on the membrane as well as any inherent biases in ligations, reverse transcriptase-polymerase chain reaction, gel migration and transfer steps (Van Nostrand *et al.*, 2016). Thus, when determining binding distributions, it is crucial to consider the background signal.

Here, we present Metadensity, a python package that supports multiple types of metagene plots and allows user-customized feature creation. In addition, it has a built-in normalization procedure to account for background in the SMInput library. Finally, it allows the user to not only utilize the read coverage as an approximation of binding, but also support the extraction of various diagnostic signals such as CITs and CIMs.

## 2 Overview

**Fig. 1.**
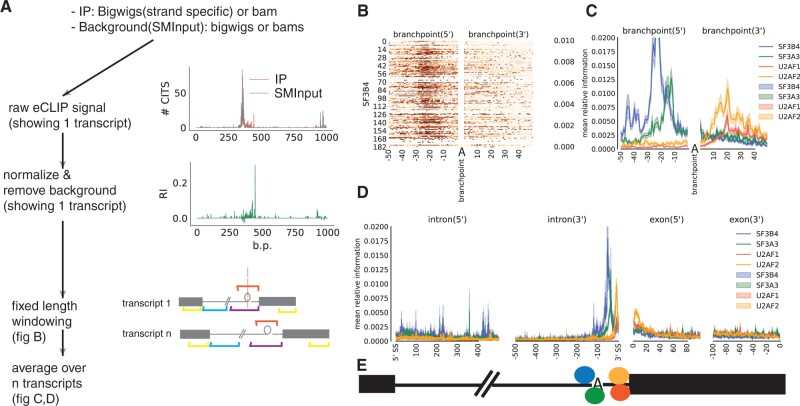
(A) Schematic overview of Metadensity steps. Top panel show the raw eCLIP signal with the number of crosslinking-induced read truncations (CITS) in IP and SMInput library. Note the peak in IP coincides with SMInput. The middle panel shows the relative information (RI) after normalizing for SMInput. Bottom panel shows how 2 fixed length windows are generated for each transcript. (B) RBP maps displaying relative information for SF3B4 around branchpoints. Each row corresponds to a transcript. Position 0 at X-axis corresponds to the branchpoint adenosine(A) sequence. The U2 snRNA is complementary to the regions surrounding A, which bulges out and attacks the 5’ss (splice site) results in lariat formation. (C,D) Median relative information around branchpoint and splice sites for SF3A3, SF3B4, U2AF1 and U2AF2. (E) Cartoon showing the underlying binding sites of the 4 proteins, based on C,D. “A” denotes the branchpoint

Metadensity starts by extracting CLIP diagnostic signals from BAM/BIGWIG files for each transcript, using either the read coverage or summation of CITs and CIMs. Alternatively, to speed up computation, a WIG track can be pre-computed (Fig. 1A), which allows us to accommodate other sequencing technologies that have signals and backgrounds in the format of BIGWIGs. The software package performs transcript-level normalization by calculating the relative information comparing IP to SMInput (Fig. 1A, middle) (Van Nostrand *et al.*, 2020). For each nucleotide of the transcript, relative information content represents the transcript-level enrichment of IP signal over the background (SMInput). Specifically, this value encodes the relative entropy that reflects the contribution of each nucleotide (see Supplementary Methods). Lastly, users can define the length of a ‘fixed window’ to extend from the 5′- and 3′-boundary of a transcriptomic feature. The relative information content values are extracted for each ‘window’ for further analysis or visualization (Fig. 1B). Metadensity outputs RBP maps (Fig. 1B), which contains the values for each individual transcript, or the mean/median across all transcripts (Fig. 1C).

The package allows users to input customized, non-Gencode features. For example, in Figure 1B–D, the metagene is supplied with branchpoints detected by CaptureSeq (Mercer *et al.*, 2015; Signal *et al.*, 2018). With this feature, we clearly recapitulate SF3B4’s role in branchpoint recognition (Brosi *et al.*, 1993; Krämer *et al.*, 1987; Moore and Sharp, 1993). Similarly, proteins part of the U2 complex has strongest enrichment at the 3′-ss. In addition, it can compute the regular 5′-UTR-CDS-3′-UTR model (Supplementary Fig. S1), and densities around polyadenylation sites (Supplementary Figs S2 and S3).

## 3 Conclusion

Here, we provide a user-friendly package to generate various metagene plots for visualizing CLIP-seq data, including pre-mRNA features such as branchpoints and polyadenylation sites. The package takes outputs from the eCLIP pipeline, fetches diagnostic signals, performs background normalization and outputs RBP maps for transcriptome-wide eCLIP visualization. Users can utilize these visualizations to interrogate RBP functions. We showcase how the U2 and SF3B complex’s density align with current knowledge and their role in the spliceosome. Similarly, U2 proteins have strongest binding at the 3′-ss. The various metagene models will allow us to propose testable hypotheses for RBPs on their impact in various steps of RNA-processing.

## Funding

This work was supported by US National Institutes of Health research grants HG004659 and HG009889.

*Conflict of Interest*: G.W.Y. is a co-founder, member of the Board of Directors, on the SAB, equity holder, and paid consultant for Locanabio and Eclipse BioInnovations. G.W.Y. is a visiting professor at the National University of Singapore. G.W.Y.’s interests have been reviewed and approved by the University of California, San Diego in accordance with its conflict-of-interest policies. The authors declare no other competing financial interests.

## Supplementary Material

vbac083_Supplementary_Data

## Data Availability

All ENCODE eCLIP datasets are available through the ENCODE website (encodeproject.org/). Annotations of transcriptomic features are available at GENCODE (https://www.gencodegenes.org/).
