# Supplementary material for: Metadensity: a background-aware python pipeline for summarizing CLIP signals on various transcriptomic sites
Source: Bioinform Adv. 2022 Nov 10;2(1):vbac083. doi: 10.1093/bioadv/vbac083 (PMC9653213; doi:10.1093/bioadv/vbac083)
Supplement: vbac083_Supplementary_Data [file vbac083_supplementary_data.docx]

## Supplementary methods

### Data availability

All ENCODE eCLIP datasets are available through the ENCODE website([encodeproject.org/](http://encodeproject.org/)). Annotations of transcriptomic features are available at GENCODE(<https://www.gencodegenes.org/>). Other transcriptomic features are available at individual databases or publications.

## Methods

### **Building Metagene and custom features**

The software starts by extracting Gencode (Harrow *et al.*, 2012) genomic features and organizes these into a transcript or gene-based dictionary. Each genomic interval (e.g. 3′ UTR) is assigned to the overlapping gene or transcripts. Additional features such as polyadenylation signals (Herrmann *et al.*, 2020), branchpoints(Mercer *et al.*, 2015; Signal *et al.*, 2018) are built into the gene-based dictionary. User can also build custom features, such as intronic polyadenylation sites, into the metagene.

### **Branchpoint Annotations**

The branchpoint annotations used in this software contain two sources. The first set (as shown in the publication) of branchpoints are detected with CaptureSeq, which captures lariat sequences with high throughput sequencing technology (Mercer *et al.*, 2015). This set of branchpoint annotation is considered high-confidence, but are only covering the genes that are highly expressed in the specific cell line used in the experiment. Therefore, we supplement the annotations with a second set of machine-learning predicted branchpoints (Signal *et al.*, 2018), and users can access it with feature ‘branchpoint-pred’. We incorporated the branchpoint adenosine(A) sequence position annotated by both methods as position 0 for windows surrounding branchpoints.

**Precomputing CLIP diagnostic signals wigs**

The entire eCLIP read represents the RBP-protected fragment. Read coverage can be computed using bedtools genomecov (bedtools: a powerful toolset for genome arithmetic — bedtools 2.30.0 documentation), then bedgraph format is converted to bigwig file using ucsctools bedGraphToBigWig. Truncation(CITS) and mutation(CIMS) are considered to be crosslinking sites, based on different reverse transcription condition. CITS is defined as the 5′ end of read, and can be extracted using bedtools genomecov with the option -5 to extract the 5′ end. CIMS can be extracted using bcftools/samtools pileup followed by a custom script CountMutations.py. A snakemake pipeline to supply precomputed tracks are available at <https://github.com/algaebrown/make_track/tree/master>. Alternatively, the package can fetch CITS signals in line using samtools/pysam(Li *et al.*, 2009).

**Background normalization**

The package offers 3 ways to consider background: Subtraction, Relative information and not considering background at all. We recommend using relative information (Van Nostrand *et al.*, 2020) (RI) because it contains less bias to coverage and RNA expression. Here we redefined the relative information used in (Van Nostrand *et al.*, 2020) to preserve nucleotide resolution, where each “element” in the original paper now represents a single nucleotide. Denote a $n$ nt transcript with any CLIP signal (CIMS, CITS or coverage) as a vector precentage of signal at the nucleotide. Denote IP library as $P$, and the SMInput library as $Q$. $P_{i}$ represents the fraction of reads covering/truncating/mutating at nucleotide $i$ in the IP library within the transcript. $Q_{i}$ is similarly defined, in the SMInput library.

Relative entropy (RI) is calculated as:

$$RI=\sum_{i}^{n} P_{i}log\frac{P_{i}}{Q_{i}}$$

To estimate the contribution of each nucleotide to RI, the normalized signal $N$ is computed as

$$N_{i}=P_{i} log\frac{P_{i}}{Q_{i}}$$

for each nucleotide $i$.

This value, $N$ is used for feature windowing and subsequent analysis.

**Windowing features**

Lastly, the software creates windows of designated feature in each transcript. For each feature, 2 fixed length windows are created aligning at the 5′ and 3′ end. If the feature occurs with multiple copies in the transcript (ex. exon), then all copies are averaged. For example, a transcript $j$ with 2 exons are at position(100,300), (900,1200) (position 0 is the 5′ transcription start site(TSS)). Let window length be $w$. The 5′ window for exon $E_{5j}$ ( a vector of length $w$ ) is calculated as: ($N\left[ 100:100+w \right]+ N[900:900+w])/2$. 3′ window$E_{3j}$ is calculated as ($N\left[ 300-w:300 \right]+ N[1200-w:w])/2$.

The window length can be customized in the config file to decide how far into the feature the user wants to visualize. It will not affect the calculating of $N$.

**Summary statistics over many transcripts**

Finally, the software calculates the mean or median over all transcripts included (Figure 1C,E). In particular, say $Z$ transcripts are included, $E_{5}=\sum_{j=0}^{Z} \frac{1}{Z}E_{5j}.$ Alternatively, users can visualize individual transcripts with plot_rbp_map() function, where a heatmap of ${{[E}_{51,}E_{52},\ldots\ldots E_{5j}\ldots E_{5Z}]}^{T}$ is shown.(Figure 1B).

### Other Metagene models

Metadensity supports other metagene models such as the traditional UTR-CDS model (Supplementary Figure 1), as well as showing densities around polyadenylation sites and signals. (Supplementary Figure 2,3)

## Optimal diagnostic signals to use

CLIP has various “diagnostic signals to use, including coverage, read truncations (CITs) and mutations (CIMs). The reliability and resolution of each signal heavily relies on the experimental protocol. Therefore, we enable options for users to decide the appropriate diagnostic signals to use. In particular, the ratio CIMS or CITs depend heavily on reverse transcription (RT) conditions [(Van Nostrand et al., 2017)](https://www.zotero.org/google-docs/?Nu1gQR). The RNase used in digestion, also affects the 3’ end formation (Haberman *et al.*, 2017), affecting the resolution offered by coverage-based metrics. we compared the binding pattern in SF3B4 (the same dataset used in figure 1) using CITS+CIMs, CIMs alone and CITs alone (Supplementary Figure 5). We noticed CIMs are sparser compared to CITs, resulting in lower magnitude of mean relative information. This is because the eCLIP protocol uses SuperScript III (SS III), which favors CITs over CIMs at crosslinking sites [(Blue *et al.*, 2022; Van Nostrand *et al.*, 2017)](https://www.zotero.org/google-docs/?v8juk9). Although both CIMs and CITs peak at -25 nucleotides 5′ to branchpoints, CITs has a trailing signal 5′ to the peaking signal, whereas CIMs has a higher smaller peak at 3′. This analysis reveals potential 5′ bias when using CITS, and a 3′ bias when using CIMs. The bias stems from the generative process of CIMs and CITs.

## Comparing metagene density between two proteins.

The values of relative enrichment over SMInput in eCLIP is driven by a few technical factors. First, the SMInput is composed of all the other RBP in the same size range. The strength of enrichment depends on the abundance of the RBP relative to the other RBPs. Second, the enrichment also depend on the quality of antibody and pull down. Therefore, when comparing two RBPs’ metagene profile, we recommend two empirical approaches: the user can either normalize the vector, or use unit-independent distance metric such as correlation distance. That way, the distance between two RBPs will be driven mostly by the relative binding signal instead of the above technical factors.

## Minimal requirements to use Metadensity.

The *optimal* requirement to use this software calculated are CLIP and SMInput bigwig files, preferably strand-specific signals (positive.bw and neg.bw for CLIP and positive.bw negative.bw for SMInput). If the technology is not strand-specific, point both of the strand-specific bigwigs to the same file.

The bare *minimum* for technologies without background controls are the bigwig file(s) from the IP. However, without the background (SM-INPUT, or IgG control) bigwigs, the package cannot calculate relative entropy and the output can be biased, and is not recommended. The user can set “background_method = None” to omit the step of calculating relative information, as shown in this tutorial [<https://metadensity.readthedocs.io/en/latest/1_Example_on_Par-CLIP-YBX1.html>]. In future releases, we hope to make this more explicit and thoroughly tested.


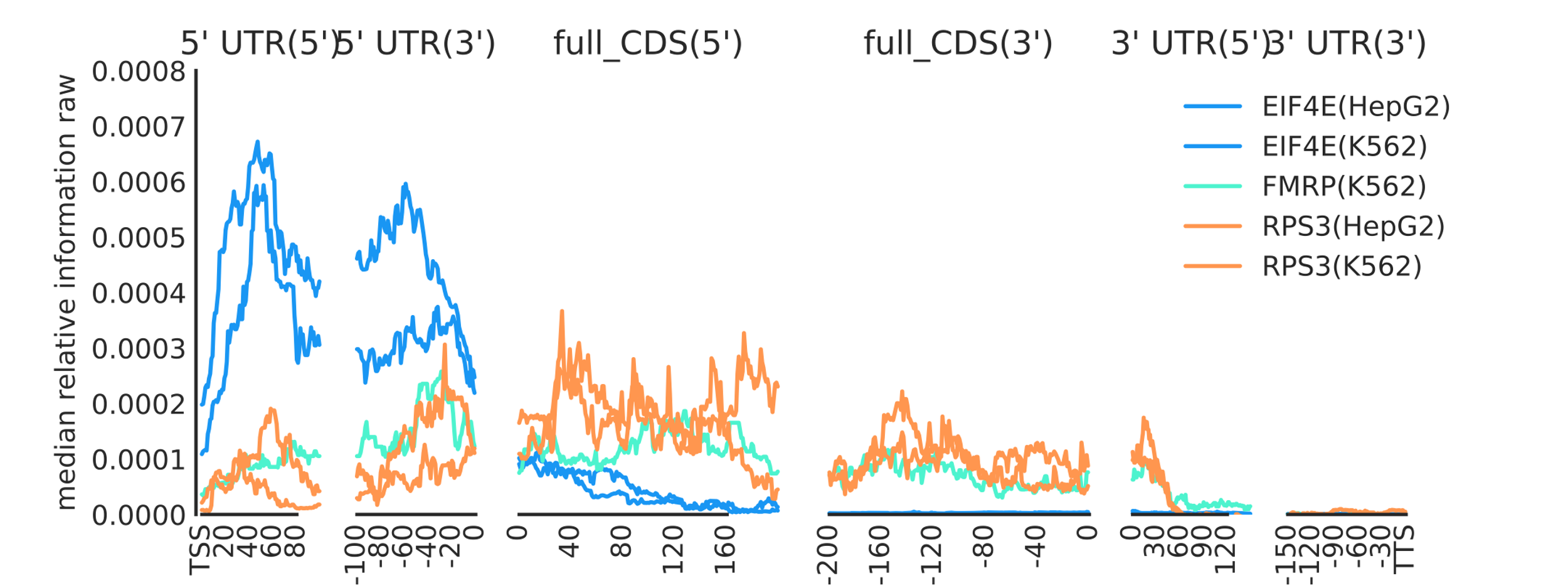


**Supplementary Figure 1: UTR-CDS-UTR model.** 5’ UTR, 3’ UTR and CDS regions are extracted from GENCODE annotations, and consecutive CDS regions are concatenated into the full CDS region. The y-axis denote median relative information (IP normalized to SMINPUT) across hundreds of highly expressed transcripts. EIF4E, a translation initiation factor, has most binding in the 5’UTR, while the ribosome (RPS3, RPS11) and its associate protein (FMRP) as more binding across the entire CDS region.


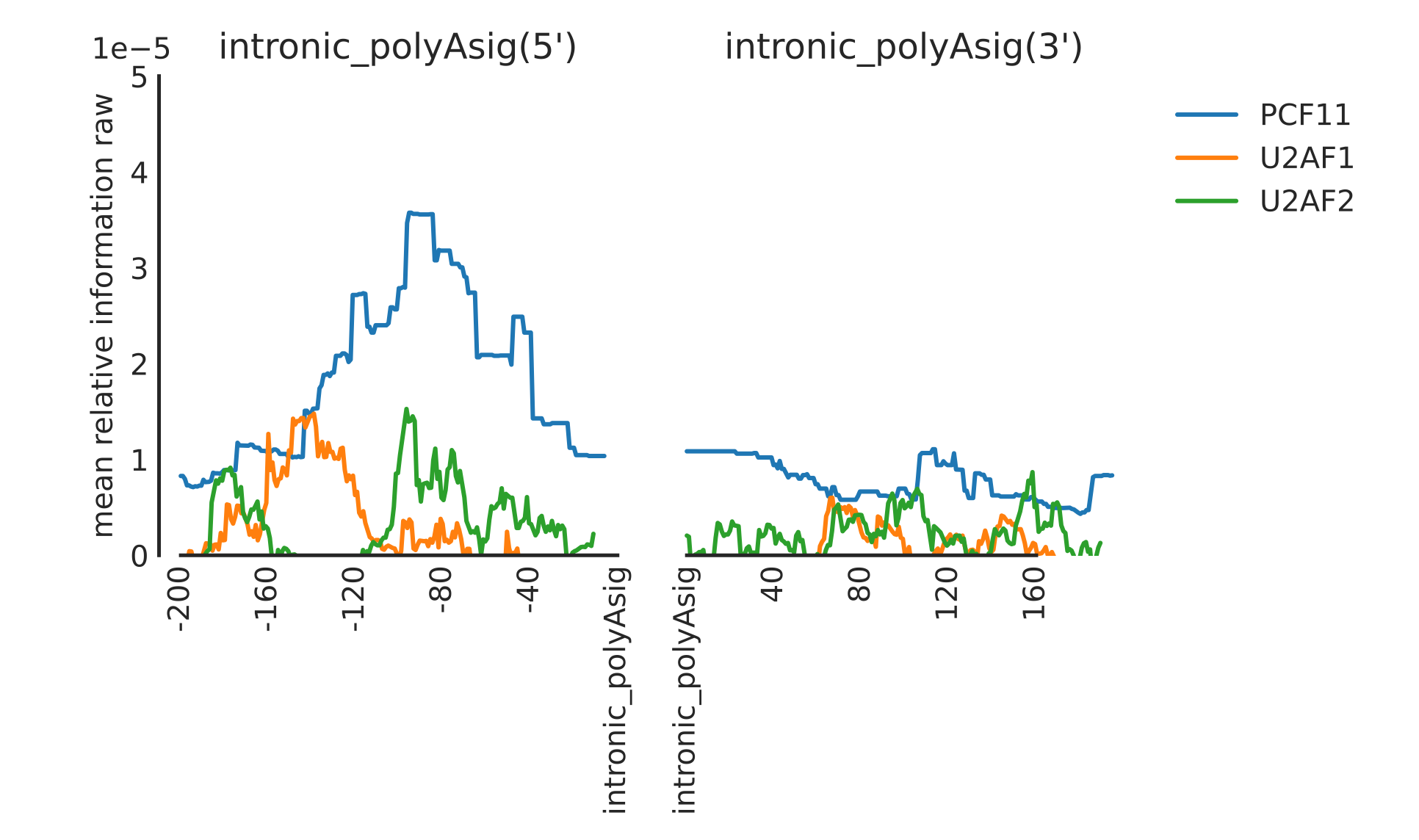


**Supplementary Figure 2 Intronic polyadenylation:** Intronic polyadenylation signals (intronic_polyAsig) are fetched from polyA Site 2.0. Y axis shows the median relative information across hundreds of highly expressed transcripts. Here we slice a 160 bp window around the annotated polyadenylation site. PCF11, cleavage and polyadenylation subunit has binding around intronic polyadenylation sites.


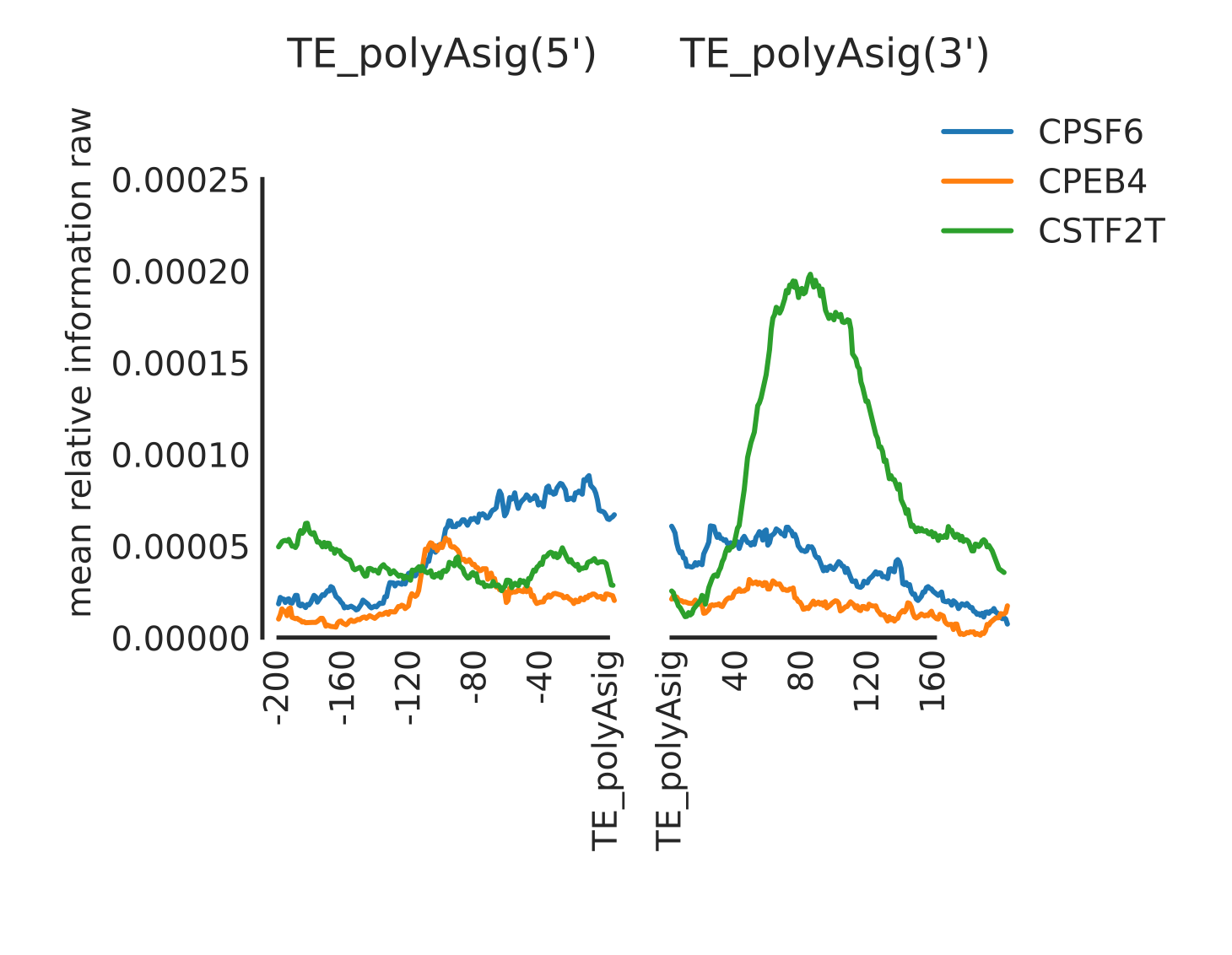


**Supplementary figure 3 Terminal exon polyadenylation:** Terminal exon polyadenylation site (TE_polyAsig) are downloaded from polyA Site 2.0. Y axis shows the mean relative information across hundreds of highly expressed transcripts. CSTF2T has binding downstream of the polyA signal, while CPSF6 and CPEB4 binds primarily upstream.


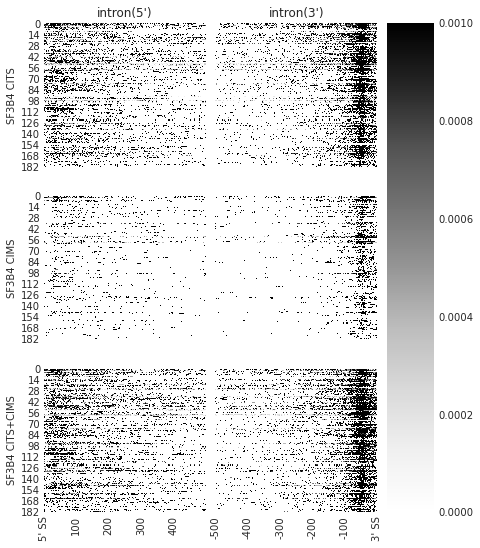


**Supplementary figure 4 RBPmaps comparing CIMs, CITs and using both signals:** RBPmaps using CITs alone, CIMs alone or using both signals (as in figure 1).


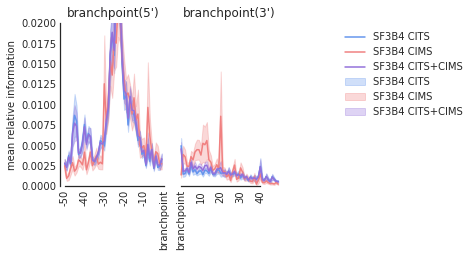


**Supplementary figure 5 Metadensity using different CLIP diagnostic signals:** CITs reveals a 5’ bias while CIMs reveal a 3’ bias.


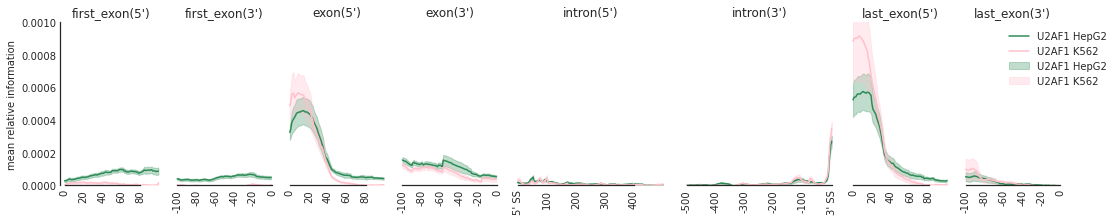
**Supplementary figure 6 Metadensity comparing U2AF1 in different cell lines**


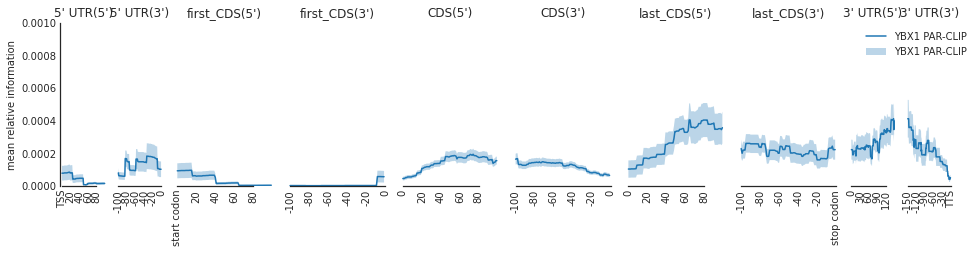


**Supplementary figure 7 Metadensity analysis on PAR-CLIP datasets(GSE150925,** (Kloetgen *et al.*, 2020, 1)**):** YBX1 preferentially binds 3’ UTR

bedtools: a powerful toolset for genome arithmetic — bedtools 2.30.0 documentation.

Haberman,N. *et al.* (2017) Insights into the design and interpretation of iCLIP experiments. *Genome Biol.*, **18**, 7.

Harrow,J. *et al.* (2012) GENCODE: the reference human genome annotation for The ENCODE Project. *Genome Res.*, **22**, 1760–1774.

Herrmann,C.J. *et al.* (2020) PolyASite 2.0: a consolidated atlas of polyadenylation sites from 3′ end sequencing. *Nucleic Acids Res.*, **48**, D174–D179.

Kloetgen,A. *et al.* (2020) YBX1 Indirectly Targets Heterochromatin-Repressed Inflammatory Response-Related Apoptosis Genes through Regulating CBX5 mRNA. *Int. J. Mol. Sci.*, **21**, 4453.

Li,H. *et al.* (2009) The Sequence Alignment/Map format and SAMtools. *Bioinforma. Oxf. Engl.*, **25**, 2078–2079.

Mercer,T.R. *et al.* (2015) Genome-wide discovery of human splicing branchpoints. *Genome Res.*, **25**, 290–303.

Signal,B. *et al.* (2018) Machine learning annotation of human branchpoints. *Bioinformatics*, **34**, 920–927.

Van Nostrand,E.L. *et al.* (2020) Principles of RNA processing from analysis of enhanced CLIP maps for 150 RNA binding proteins. *Genome Biol.*, **21**, 90.
